# Biotechnological Perspectives to Combat the COVID-19 Pandemic: Precise Diagnostics and Inevitable Vaccine Paradigms

**DOI:** 10.3390/cells11071182

**Published:** 2022-03-31

**Authors:** Mahender Aileni, Gulab Khan Rohela, Phanikanth Jogam, Shakuntala Soujanya, Baohong Zhang

**Affiliations:** 1 Department of Biotechnology, Telangana University, Dichpally, Nizamabad 503322, India; 2 Central Sericultural Research & Training Institute, Central Silk Board, Pampore 192121, India; gulab_biotech@yahoo.co.in; 3 Department of Biotechnology, Kakatiya University, Warangal 506009, India; phanikanth.jogam@gmail.com; 4 Department of Oral Medicine and Radiology, Meghna Institute of Dental Sciences, Nizamabad 503003, India; shakuntalasoujanya@gmail.com; 5 Department of Biology, East Carolina University, Greenville, NC 27858, USA

**Keywords:** ARDS, coronavirus, detection methods, SARS-CoV-2, COVID-19, CRISPR, vaccine, nucleic acid test (NAT)

## Abstract

The outbreak of the novel severe acute respiratory syndrome coronavirus 2 (SARS-CoV-2) is the cause for the ongoing global public health emergency. It is more commonly known as coronavirus disease 2019 (COVID-19); the pandemic threat continues to spread aroundthe world with the fluctuating emergence of its new variants. The severity of COVID-19 ranges from asymptomatic to serious acute respiratory distress syndrome (ARDS), which has led to a high human mortality rate and disruption of socioeconomic well-being. For the restoration of pre-pandemic normalcy, the international scientific community has been conducting research on a war footing to limit extremely pathogenic COVID-19 through diagnosis, treatment, and immunization. Since the first report of COVID-19 viral infection, an array of laboratory-based and point-of-care (POC) approaches have emerged for diagnosing and understanding its status of outbreak. The RT-PCR-based viral nucleic acid test (NAT) is one of the rapidly developed and most used COVID-19 detection approaches. Notably, the current forbidding status of COVID-19 requires the development of safe, targeted vaccines/vaccine injections (shots) that can reduce its associated morbidity and mortality. Massive and accelerated vaccination campaigns would be the most effective and ultimate hope to end the COVID-19 pandemic. Since the SARS-CoV-2 virus outbreak, emerging biotechnologies and their multidisciplinary approaches have accelerated the understanding of molecular details as well as the development of a wide range of diagnostics and potential vaccine candidates, which are indispensable to combating the highly contagious COVID-19. Several vaccine candidates have completed phase III clinical studies and are reported to be effective in immunizing against COVID-19 after their rollout via emergency use authorization (EUA). However, optimizing the type of vaccine candidates and its route of delivery that works best to control viral spread is crucial to face the threatening variants expected to emerge over time. In conclusion, the insights of this review would facilitate the development of more likely diagnostics and ideal vaccines for the global control of COVID-19.

## 1. Introduction

The current viral outbreak of the SARS-CoV-2 infection spreading COVID-19 was originally reported in Wuhan, China (December 2019). The life-threatening acute respiratory distress syndrome (ARDS) is a prominent symptom of COVID-19 [1].This is a pandemic that is threatening public health and has spread over the globe very quickly [2,3]. In view of SARS-CoV-2’s contagious and fast-spreading tendency, its outbreak has been detected in over 220 countries. Owing to this, 6.00 million people have died, and 442.33 million people have tested positive for the virus as on 4 March 2022 [4]. In the process of preventing SARS-CoV-2outbreaks, quarantine protocols, which have been imposed from time to time, have drastically affected socio economic well-being [5]. The coronavirus disease 2019 (COVID-19) is known to be the world’s first severe, highly contagious, and deadly pandemic of the twenty-first century [6,7].

Coronaviruses (CoVs) have a positive-sense single-stranded (ss) RNA genome (size: ~29.9 KB) enclosed in a capsid protein that is comparatively larger than other viruses. When seen under an electronic microscope, projections of glycoproteins appear as spikes on the envelope, giving it a crown-like appearance [8,9]. Coronaviruses (CoVs) are divided into four genera, namely, AlphaCoV, BetaCoV, DeltaCoV, and GammaCoV. The class of beta coronavirus includes the Middle East respiratory syndrome (MERS) virus, severe acute respiratory syndrome virus (SARS-CoV), and the SARS-CoV-2, the causative agent of COVID-19. Similar to SARS-CoV and MERS-CoV, the SARS-CoV-2 virus infects by binding to angiotensin-converting enzyme-2 (ACE2) receptors [8,10] present on the gastrointestinal system, lower respiratory system, liver, central nervous system, kidney, and heart. Initially, virus-infected cells can readily escape the host’s interferon, which results in greater virus replication in the lungs and leads to the release large levels of pro-inflammatory cytokines. This is known as a “cytokine storm”, which causes inflammation-related lung injury. Death is caused by the failure of multiple organs, which is most common in elderly persons with co-morbidities [11]. The current grim situation is shaking the global healthcare system due to the elevated SARS-CoV-2 infectious nature and mortality rate in humans [4].

In view of the trend in the rapid spread of SARS-CoV-2, it is crucial to break the human-to-human viral chain of transmission under containment and management strategies of COVID-19 [12]. Corona infection spreads directly via larger respiratory droplets to small aerosols or indirectly through contaminated surfaces, which is quite similar to how common cold and influenza viruses spread. The COVID-19 virus has a basic reproductive potential (RO) of 3.8, which is defined as the number of cases directly transmitted by one infected person in a population. This value is known to be much higher than estimates by the WHO, with an RO value of 1.3 to 2.5 [13]. The rate of transmission of MERS-CoV and SARS-CoV are relatively lower as indicated by their lower RO values (i.e., <1.0) [14]. SARS-CoV-2 infections, with an RO value indicating a higher infection rate (i.e., 3.28), have increased due to the fact of asymptomatic transmission and longer incubation periods [15]. This implies that preventing and controlling SARS-CoV-2 infection will be challenging [16,17]. Thus, the major challenge for preventing the spread of the COVID-19 virus is identifying and isolating asymptomatic people as they are the unknown source of transmission [18]. Undiagnosed and asymptomatic cases continuously increase the risk of spreading COVID-19 infection [19,20]. Further, half of the COVID-19 cases are asymptomatic, as they do not show signs of severe illness before admission to hospitals [21].

Moreover, due to the fact of its high prevalence, wider distribution, profound genetic diversity, higher potential of genomic recombination, and reported human–animal transmission cases, the COVID-19 pandemic is considered a health threat at present and in the future [17].Thus, the more the virus is allowed to spread (i.e., leap species to species), the more opportunity the virus accumulates to mutate during its multiplication, which facilitates the virus to become more or less deadly or change its receptor-binding domain, which would potentially interfere with therapeutic or vaccine development. There is an urgent need to monitor more fit variants of disease transmission across populations and geography in a quick and real-time manner, since they pose a potential public health threat [22]. The fatal 2nd and 3rd surge of COVID-19 spread in highly populous countries, including India, Brazil, and the United States, signifying a prevailing uncontrolled disaster trend [23,24,25].

The development of precise and rapid detection tests plays a crucial role in containing the spread of highly contagious diseases. Currently, SARS-CoV-2 virus-based COVID-19 detection in humans is being tested in a variety of methods in different countries [26,27]. Due to the urgent public health emergency, unprecedented efforts were made around the globe for the ready diagnosis of SARS-CoV-2 at the individual level and its outbreak status at the community level [28,29,30]. Cost-effective and POC-based diagnostic methods are the critical need of the hour to understand COVID-19 epidemiology, contact tracing, and case management [31]. Thus, wide-spread deployment of accurate and early diagnostic tests of viral-specific antigen, antibody titers, and nucleic acids will help to identify the SARS-CoV-2 stage of infection, immediate isolation, and its containment strategies [32,33]. Quantitative real-time reverse transcription-polymerase chain reaction (qRT-PCR) assays remain a prime molecular test and cornerstone of COVID-19 testing [33,34]. In addition, various approaches are being used to diagnose COVID-19 including isothermal nucleic acid amplification assays, CRISPR-based analysis, and hybridization microarray assays [30,35,36]. Development of plug-and-play diagnostic methods aid in timely disease control by screening emerging SARS-CoV-2 variants; thus, such detection methods play pivotal role in averting future pandemics.

Given the unavailability of a successful antiviral drugs for COVID-19 treatment [31,37], current case management comprises early and rapid detection, urgent isolation of positive cases, general supportive care, respiratory support, and nutritional support. Vaccine development represents a human adaptive strategy used to prevent and control viral outbreaks (measles, virus hepatitis A, poliomyelitis, mumps, and rubella); thus, vaccines against SARS-CoV-2 are considered the ultimate intervention to contain the COVID-19 pandemic [38]. Vaccines train and prepare the body’s natural defenses to identify and destroy disease-causing foreign agents. If vaccinated people are later exposed to the same disease-causing agents, their body system induces effective immunity (secondary immune response) against the pathogens through activation of cellular (T cell) and humoral (antibody) immune responses. Thus, vaccines are prophylactic in nature and develop adaptive immunity; therefore, they save millions of lives every year through immunization campaigns. In the process of preparedness efforts, several nations are racing to deploy safe and efficient vaccines to combat the SARS-CoV-2virus and its associated morbidity and mortality [39]. In order to combat COVID-19 disease, several vaccine candidates are in the development pipeline using a variety of platforms including some based on viral vectors (non-replicating and replicating viral), recombinant peptide/protein subunit/virus-like particles (VLPs), nucleic acid (RNA and DNA), and whole virus (inactivated or attenuated) [40,41]. Safe and effective vaccines will be a game-changer: vaccine-induced immunity is a more potent public tool for preventing virus reproduction and transmission [22]. Several vaccine candidates have completed phase III clinical trials and have been given EUA for massive and accelerated vaccination campaigns. As a result, the vaccination process covers the population’s protective proportion—herd immunity—which is instrumental for COVID-19 disease prevention [42,43]. However, choosing the type of vaccine and the most appropriate delivery strategy (vaccination route) would be decisive for improving vaccine efficacy against current or emerging new variants of the coronavirus.

In this COVID-19 pandemic scenario several studies have been performed to immediately control and prevent virus infection and outbreak [44]. In this context, a review paper with current findings on diagnostic tests and vaccine paradigms could speed up future COVID-19 infection containment and prevention studies. Emerging biotechnologies largely contribute to fighting this invisible enemy, COVID-19, and more importantly on fronts of its diagnosis and vaccine development. Accordingly, this review aims to present an overview of recently developed diagnostic tests for SARS-CoV-2 and vaccination candidates that have been rolled out to immunize human’s against COVID-19.

## 2. Diagnostics

Diagnostic tests that are both robust and precise are pivotal for determining the infectious state of an illness. Tests of diagnosis are essential for identifying disease severity, and the prognosis is what follows the course of that disease and its treatment. Diagnostics with improved sensitivity and specificity at the individual to community levels would prevent false-positive or false-negative testing of COVID-19 infection. This is the need of hour, as this precision testing not only facilitates to reveal the status SARS-CoV-2 outbreak but also aid in its rapid containment and management.

Since the first cases of COVID-19 pandemic reported, due to more suitable mutational changes in the genome of SARS-CoV-2, there has been an escalating rate of its potential virulent strains. It emphasizes the importance of accurate and rapid diagnosis of asymptomatic cases, immediate symptomatic treatment, large-scale immunization campaigns, and practicing of COVID-19 appropriate protocols. These measures would only be the keys to successful management of the COVID-19 disease. The current contest to develop low-cost point-of-care (POC)-based diagnostic kits and laboratory-based methods for precise and/or early confirmation of SARS-CoV-2 virus infection has sparked a new wave of diagnostic development (Table 1). POC assays are those detection methods that enable the processing of patients’ clinical samples without the use of centralized laboratory equipment. Such assays give a COVID-19 positive or negative test result for the patient in less than one hour and even in minutes.

Basically, the COVID-19 diagnostic methods fall into two major categories: Molecular tests (direct methods) are used to detect viral genetic material (RNA) of SARS-CoV-2 using nucleic acid hybridization methods or by polymerase chain reaction (PCR) techniques. Immunological and serological assays (indirect methods) are the second category, which are used to evaluate antibody titers or detect viral antigenic proteins in individuals. Direct methods, detecting SARS-CoV-2 viral RNAs, are used to identify virus-infected people who are either asymptomatic or at the severe phase of COVID-19 infection. These methods are applied not only for contact tracing studiesbut also help to detect time-to-time emergenceof SARS-CoV-2 variants [54]. When methodically applied, contact tracing aidsintracing the chain of contagious disease transmission and is considered as anessential tool to control disease outbreaks. In contrast, indirect methods check the immunity status of individuals and communities over a period of time [52,56,57]. As a part of this review article, we reported current SARS-CoV-2 detection trends based on conventional and existing methodologies, which may assist innovations in the field of disease diagnostics.

### 2.1. Reverse Transcription-Quantitative Polymerase Chain Reaction (RT-qPCR)

**Figure 1:**
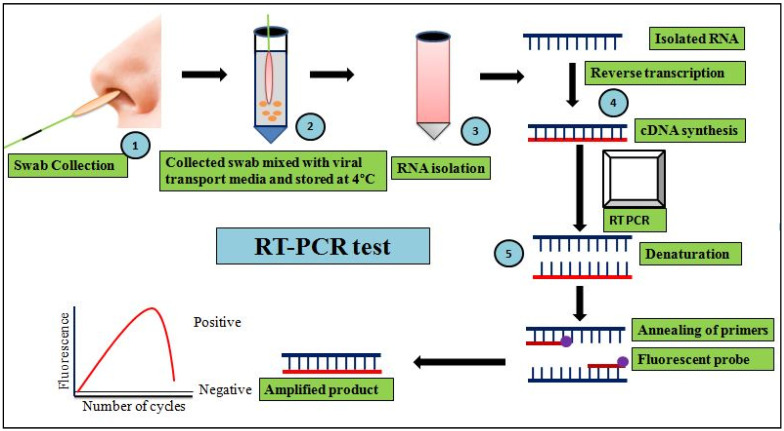
Flow chart of RT-PCR--based detection of SARS-CoV-2 infection: (**1**) collection of the test sample from anindividual; (**2**) mixing the collected sample with viral transport media and storageat 4 °C until use; (**3**) isolation of viral RNAs from the collected sample; (**4**) synthesis of cDNA from viral RNA by reverse transcription; (**5**) RT-PCR with specific primers/fluorescent markers demonstrates either positive or negative results for SARS-CoV-2 detection.

RT-PCR is one of the foremost developed gold-standard tests used to detect viral loads of SARS-CoV-2 virus in COVID-19 pandemic [58]. It works on the basis of reverse transcriptase (RT) generating complementary DNA (cDNA) from the viral RNA template, followed by 40 cycles of exponential amplification of cDNA to double-stranded (ds) DNA. Clinical samples’ RNA levels are amplified using primer-probes designed for the targeted regions of SARS-CoV-2, including *ORF1ab*, *OR1a*, envelope (E) genes, RNA-dependent RNA polymerase (RdRP), spike (S) protein, and the nucleocapsid (N) [29,58]. It is a quantitative (qPCR)-based amplification method that monitors viral load in clinical samples using fluorescent or electrical signals [59]. As a result, the viral load in the form of cyclic threshold (Ct) values are determined by quantifying SARS-CoV-2 viral RNA in clinical samples(swabs) collected (under bio-safety protection 3 level) generally from individuals’ upper respiratory tract. Clinical samples with a higher viral load indicate a low Ctvalue. The cut-off Ct value is 35, below which COVID-19 positivity is considered, and the above which COVID-19 is considered negative, while Ct values ≤ 25 indicate a higher viral load [60]. These values are used to evaluate whether a person is positive for COVID-19 and to establish the status of COVID-19 patients’ isolation periods [34]. Aflow chart of RT-PCR is depicted in Figure 1. RT-PCR kits are currently manufactured by a number of companies, allowing researchers to deal with a variety of clinical samples such as respiratory swabs, serum, saliva, stool, and ocular secretions (Table 1).For COVID-19 diagnosis, customized cartridge-based amplification of nucleic acid tests (CB-NATs) and chip-based NATs have also been used. Other advancements inmolecular diagnosis of COVID-19 include nested RT-PCR and droplet digital PCR. Nested RT-PCR is an altered RT-PCR, having the merit of increased amplification specificity by reducing altered products generation due to the fact of non-specific primer binding. This technique has a testing sensitivity of 95% using low viral load samples [58]. However, this new PCR method also hassome disadvantages including being costlier than qPCR and cross-contamination-caused false-positive results [54].

### 2.2. Isothermal Nucleic Acid Amplification

Instead of RT-PCR, isothermal nucleic acid amplification is the method which does not require thermal cycling was developed having applications in disease diagnosis. Because of this feature, it is the quickest and most convenient molecular biology detection method for diagnosing SARS-CoV-2 infection [45]. This method enables primer-mediated polymerization of the target sequences by strand-displacement activity. Based on this principle, two additional methods were developed, which include:

- Reverse transcription loop-mediated isothermal amplification (RT-LAMP);
- Transcription-mediated amplification (TMA).

#### 2.2.1. RT-LAMP

**Figure 2:**
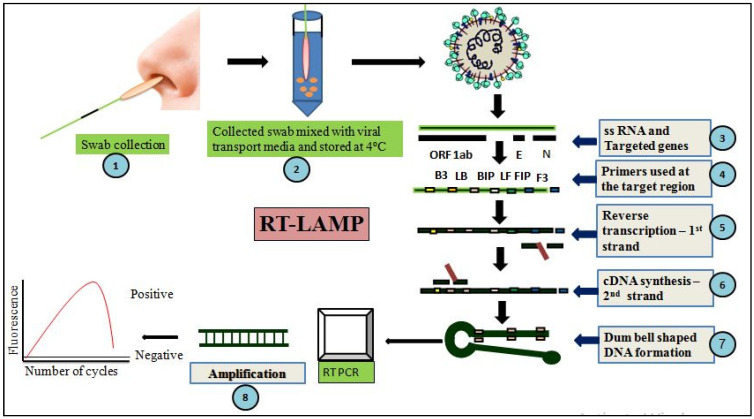
RT-LAMP technique-based detection of SARS-CoV-2 infection: (**1**) collection ofhuman samples; (**2**) mixing of the collected sample with viral transport media and storageat 4 °C until use; (**3**) single-stranded RNA genome of SARS-CoV-2 with the targeted genes; (**4**) specific primers binding atthe targeted genes; (**5**) synthesizing the first-strand cDNA by reverse transcription; (**6**) DNA polymerization for second-strand cDNA synthesis; (**7**) dumbbell-shaped DNA formation during the process of cDNA synthesis; (**8**) RT-PCR with specific primers/fluorescent markers demonstrates either positive or negative results for SARS-CoV-2 detection.

This technique is a modification of isothermal nucleic acid amplification, which is specifically used to diagnose infectious diseases caused by RNA viruses [61]. It is accomplished by the three sequential steps:

1. Reverse transcription of viral RNA template to synthesize cDNA by enzyme reverse transcriptase;
2. Bst DNA polymerase directed DNA amplification using two inner primers (i.e., BIP andFIP) and two outer primers (i.e., B3 andF3). These will prime the target cDNA to form a dumbbell-like DNA product, a prerequisite for isothermal amplification (Figure 2). Following this, spontaneous cyclic reactions of amplification give several copies of target sequences;
3. Calorimetric interpretationby photometry-based detection of the viral DNA ornaked eye visualization based on magnesium pyrophosphate-mediated precipitation reaction [62].

Thus, this technique is one of the promising tools used for the detection of COVID-19.

#### 2.2.2. Transcription-Mediated Amplification (TMA)

**Figure 3:**
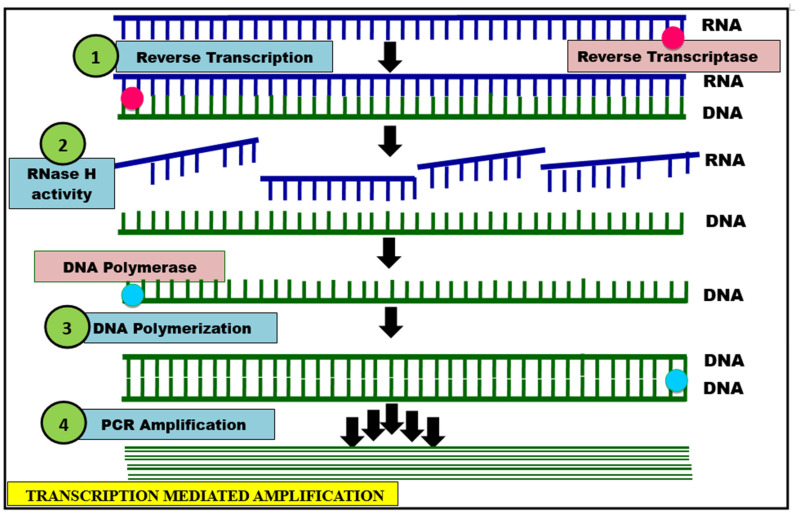
Transcription-mediated amplification (TMA)-based detection of SARS-CoV-2 infection: (**1**) reverse transcription-based first-strand cDNA synthesis; (**2**) RNA strand of hybrid RNA–cDNA is degraded by the RNase H activity of the enzyme reverse transcriptase; (**3**) DNA polymerization for the synthesis of second-strand cDNA; (**4**) PCR amplification with specific primers will demonstrate either positive or negative results for SARS-CoV-2 virus detection (multiple arrows indicate multiple cycles of amplification).

It is a single tube-based isothermal nucleic acid amplification system thatworks on the principle of both RNA transcription and cDNA synthesis using RNA polymerase and reverse transcriptase simultaneously. In this method, instead of DNA amplicon, RNA amplicon is produced from the target nucleic acidin question [63]. The TMA mechanism is initiated when the template viral RNA hybridizes with specific capture probe. Upon the addition of oligonucleotides promoter site is bound by T7 RNA polymerase, this directs the oligonucleotides to capture onto magnetic microparticles under the influence of magnetic field. There by forms an active complex of T7 promoter sequence linked primer. Wherein the RNA molecule formed undergoes reverse transcription producing a cDNA. Later on, during the first strand of cDNA synthesis, the RNA strand of the hybrid RNA–cDNA is degraded byRNase H activity of the enzyme, while reverse transcriptase aids in single-stranded (ss) cDNA formation. In the final step, many RNA amplicons are produced by the action of T7 RNA polymerase [30], leading to their re-entry into the TMA process. This exponential amplification generates billions of RNA amplicons in less than 1 h (Figure 3). Based on this principle, a platform called Panther fusion is used to sense ss nucleic acid detection viaa fluorophore and quencher. This platform of panther fusion is unique due to the fact of its high turnover rate (up to 1000 tests in 24 h), and this method clearly demarcates other frequent respiratory viruses causing similar symptoms of COVID-19. In addition, the availability of another version of Panther fusion, called Hologic’s Panther Fusion platform, simultaneously performs both TMA and RT-PCR for the detection of SARS-CoV-2 RNA more accurately [58,64].

### 2.3. CRISPR-Based COVID Detection Assay

**Figure 4:**
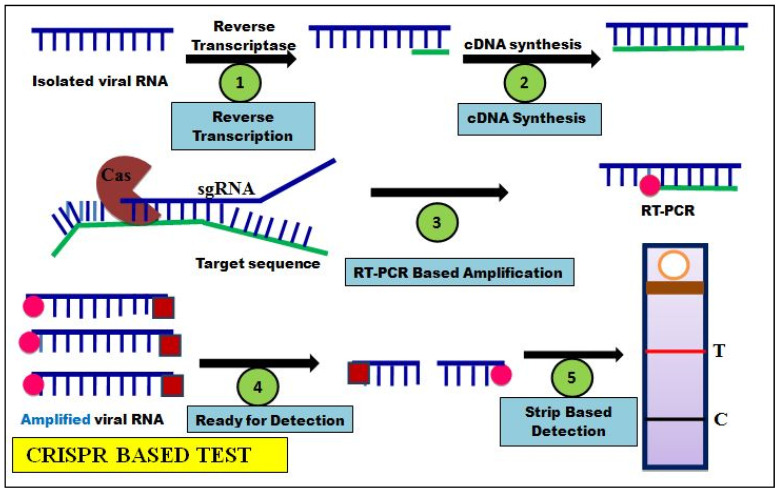
CRISPR/Cas-based detection of SARS-CoV-2 infection: (**1**) reverse transcription of isolated viral RNA; (**2**) reverse transcription for the synthesis of cDNA; (**3**) CRISPR-mediated genome editing (identify and cleave) actat the precise sequence of viral RNA SARS-CoV-2 genome. Both Sherlock and Detector of the CRISPR tool convert viral RNA to DNA (isothermal amplification), which activates nuclease enzyme activity (Cas-12/13) to cleave the target sequence (pink colored symbols indicate DNA polymerase;red colored symbols indicates primer); (**4**) loading of the sample (fluorescence RNA reporter) on to strip for detection of the specific viral RNA sequence; (**5**) the number of bands visible on the strip (lateral flow) represents whether the test is positive or negative for SARS-CoV-2 infection.

Clustered regularly interspaced short palindromic repeats (CRISPR) are edited DNA sequences that were first noticed as chopped nucleic acids in bacteriophage-infected bacteria. Its mechanism of action was discovered while studying the bacterial defense system, which demolishes the DNA of similar bacteriophages during their subsequent infections. This specific molecular mechanism understanding led to the discovery ofCRISPR and Cas enzyme (endonuclease) technology, a cutting-edge tool facilitates precision genome editing studies in various organisms [65,66]. This technology gained high popularity due to the fact of its efficiency and specificity in the genome editing process, also called targeted mutagenesis [65,67]. Both endonuclease and synthetic guide RNA (gRNA) work together to edit (insertion/deletions) a specific DNA sequence, resulting in the altered genomes of interest [68]. Thus, endonucleases, such as Cas9, cleave the targeted DNA around theprotospacer adjacent motif (PAM), which facilitates to create interestededits in different organisms for various research applications [65,69]. The CRISPR/Cas tool, which is a prominent technology, is also being used to detect viral infections in humans [70,71]. The CRISPR system uses specific endonucleases (such as Cas12a and Cas13a) and gRNA to detect the genome of viral pathogens [47,72]. This CRISPR system has now been extended for the diagnosis of SARS-CoV-2 infections in humans (Figure 4).

Specific high-sensitivity enzymatic reporter unlocking (Sherlock) and DNA endonuclease-targeted CRISPR trans reporter (Detector) are two important techniques of CRISPR for SARS-CoV-2 detection.In these techniques, instead of Cas9, either the Cas12 or Cas13 endonuclease proteins are used to cleave and identify the SARS-CoV-2 viral genetic materials [29,45]. Sherlock is a CRISPR/Cas13-based tool used to precisely detect viral RNA genome in COVID-19 patient samples [73], wherein Cas13 cuts the SARS-CoV-2 *ORF1ab* gene-targeted RNA sequence. In this process, RNA of throat/respiratory swabs/samples aresubjected to reverse transcription to synthesize cDNA based on reverse polymerase chain reaction and amplified to give products [48,54,74]. The amplified product is further transcribed into RNA amplicons. Then, specific synthetic gRNA and Cas13 complex recognize to bind the amplified RNA product [75]. The Cas13 RNA targeting enzyme activity cleaves both target and non-target nucleic acids of the patient sample. The targeted product upon binding by the fluorophore, quencher probes cleaved by activated Cas13, givea fluorescence signal [48,74]. The Sherlock technique reads out clinical samples to determine anoutcome within 1 h [76]. Gootenberg et al. (2017) [77] initially developed this Sherlock method; later, it was refined by Kellner et al. [73] to specifically detect the COVID-19 RNA genome. Due to the CRISPR multiplexing, the Sherlock method detects more than 160 differentpathogenic agentspresenting in patient samples [78]. While, CRISPR/Cas12 editing technology is used to detect the SARS-CoV-2 genome through the DNA endonuclease-targeted CRISPR trans reporter (detector) technique [79]. The fluorescent probe and CRISPR/Cas12 were employed to detect the differential RNA amplicons that can be used to confirm SARS-CoV-2. This technique is handy, as is can be conducted outside of the clinical diagnostic laboratory, indicating POC testing. The negative results of RT-PCR can be found to be positive in CRISPR-based fluorescent detection due to the fact of its accuracy [48].

### 2.4. Microarray Nucleic Acid Hybridization

RT-qPCR is the gold-standard test for the clinical diagnosis of SARS-CoV-2. But for the analysis of a large number of samples, there is a need for an approach that uses more stringent nucleic acid hybridization conditions which prevents their mismatch base pairing. Such developed methods overcome the RT-qPCR associated false-negative results during disease diagnosis [80]. Microarray nucleic acid hybridization is one of the basic fundamental molecular tests that encompass the use of single-stranded (ss) nucleic acids DNA/RNA as microscopic spots (chip), which hybridizes with cDNA prepared from the RNA genome of SARS-CoV-2. The labeled cDNA fluorescent probe identifies complementarySARS-CoV-2 RNA molecules present in the clinical samples. After washing, labeled probes that are hybridized with the specific nucleic acid of SARS-CoV-2 areexcited to produce a signal. Currently, this diagnostic approach is deployed for detecting mutations in single-nucleotide polymorphisms (SNPs) and genotyping of emerging SARS-CoV-2 variants [49,50]. As a result of its multiplexing and high specificity, DNA microarray hasbeen emerged as one of the most promising diagnostic methods for SARS-CoV-2 detection [81].

### 2.5. Genome Sequencing

**Figure 5:**
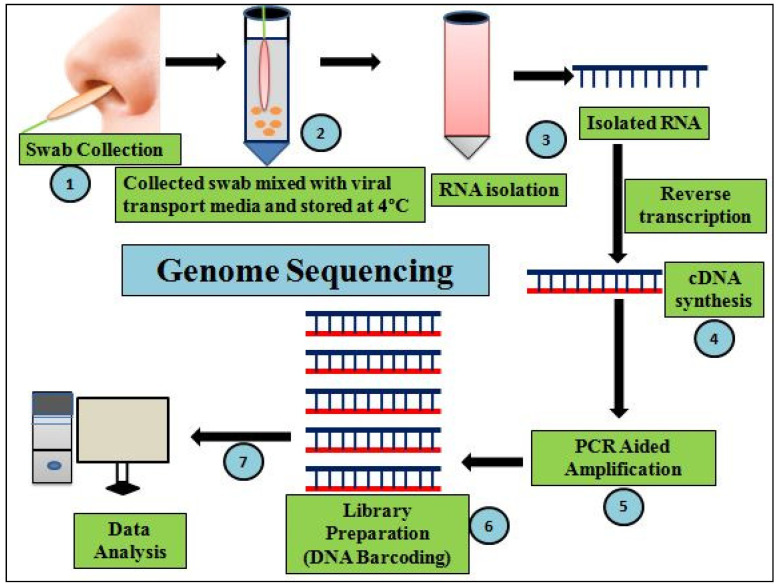
Genome sequencing for detection of SARS-CoV-2 variants: (**1**) swab/test sample collection from an individual; (**2**) mixingthe collected sample with viral transport media and storage at 4 °C; (**3**) isolation of viral RNA from the collected sample; (**4**) cDNA synthesis from viral RNA by reverse transcription; (**5**) PCR-aided amplification; (**6**) computer-aided library preparation and collection of nucleotide databases; (**7**) further data analysis: RNA/whole-genome sequencing via NGS to identify SARS-CoV-2 variants assist in redesigning novel molecular-based diagnostics therapies to combat COVID-19.

Genomic sequencing tools are aversatile platform withimplications in different scientific fields such asagriculture, public health interventions, pathogen origin, contagious disease outbreaks, and phylogenetic analysis [82]. In the process of being prepared for future public health threats, current whole genomic sequencing trends need to be explored in every COVID-19 affected nation at an accelerated rate to build a healthy global community. High throughput genomic analysisvianext-generation sequencing (NGS) ensures the identification of novel pathogens of evolutionary/zoonotic origins and their rate of mutation or recombination frequency over time [83]. Investigations on genomics and molecular epidemiology of a disease organism (2019 novel coronavirus) unravel the origin and receptor binding of host–pathogen during itsinfection process. Such investigations are the real-time basis for designing novel molecular-based diagnostics and therapies required to control pandemics [84]. Rapid SARS-CoV-2 RNA isolations, differential RNA-Seq, library preparation protocols, identification of genomic sites of antiviral resistance, and deposition of repository genomic/protein/nucleotide sequence databases are used for scaling time-to-time viral outbreak intensities [82,85]. The due emergence of a given virus with a different genomic sequence, when it is tested with the existing probe results in a false–negative outcome leads to non-diagnosis of COVID-19 infections. In view of the emerging SARS-CoV-2 variants, the diagnostic test failures need to be correlated by whole genomic sequencingto reveal insights into whether failures weredue to the fact of sequence divergence or test failures [86]. Therefore, advances in diagnostics and improved genomic surveillance are two goals that need to work hand in hand for examining/handlingSARS-CoV-2 transmissibility or future pandemic threats. In addition, genomic sequencing of emerging variants may not only havean impact on innovations in clinical diagnostics but could also influence real-time vaccine redesign required to immediately curtail COVID-19 infections [87]. Therefore, large-scale deployment of time-to-time viral genomic sequencing (Figure 5) is required to characterize SARS-CoV-2 variations for likely diagnosis and clinical significance.

### 2.6. Biosensors

These are the medical gadgets that are readily used to diagnose diseases ranging from acute to chronic in nature when laboratory equipment is in short supply/lacking. Thus, biosensors serve the purpose of detecting disease diagnosis within no or limited availability of resources, representing point-of-care diagnosis. Biosensors are generally classified into different types based on the type of transducer employed or the type of bioreceptor utilized. The basic type of biosensors arecomposed of gold nanoparticles (AuNP) of 20–80 nm and are precipitated with either coronavirus surface antigens or antibodies. It works on the principle of surface plasmon resonance (SPR), wherein changes such as adsorption/detection of biologically active compounds (such as antibodies/antigens/enzymes) are converted into incident light at surface interference and transducesreadoutcome via optical, electrical, and enzymatic methods [29]. Biosensors developed to diagnose severe acute respiratory syndrome (SARS) arecomposed of biochips incorporated with surface antigens that detect 200 ng/mL antibodies in a few minutes [88]. The CANARY biosensors, which were now specifically designed to detect the COVID-19 virus by PathSensors Inc., are based on a cell-based immune biosensor that transduce to give results in 3–5 min by capturing the virusviasignal amplification. Most recently, field-effect transistor (FET)-based biosensors were established for detecting the SARS-CoV-2 viral components.It is constituted of FET-based graphene sheets coated with a specific antibodiesagainst the spike protein ofSARS-CoV-2. These FET-based biosensors have a limit of detection of 2.42 × 10^2^ copies/mL in clinical samples and an LOD of 1.6 × 10^1^ pfu/mL in culture medium [89]. Apart from these, the aptamer-based biosensors were developed specifically to detect SARS-CoV-2 in asymptomatic patients. Such biosensors have the features of higher sensitivity, specificity, selectivity, and cost-effectiveness [52,54].

### 2.7. Serological and Immunological Assays

These assays are commonly used to track infectious disease outbreaks by correlating past and/or present immunity status from the individual to community levels over a period of time [90]. Government officials of a given nation conduct these tests as part of a survey to assess population/herd immunity against a given infectious disease. The data generated by such survey generally aid in the epidemiological, diagnostic, and vaccine redesigning investigations [31]. These tests examine immune status by looking for two specific antibody classes (immunoglobulin G (IgG) and immunoglobulin M (Ig M)) against the most prevalent pathogenic antigens (e.g., spike protein of SARS-CoV-2) present in clinical samples such as saliva, sputum, and blood. As a front-line defense mechanism, the human immune system soon after infection produces IgM than IgG. However, IgG, on the other hand, has a long-term immunological memory and will respond to the same pathogen if it is encountered again. IgM antibodies are an early indicator of infection, whereas IgG antibodies are a current or post-infection immunity indicator [30]. In addition, immunoglobulin A (Ig A) responses in mucosal secretions are also found at greater titers. These immunoglobulin responses have prime significance in the current SARS-CoV-2 outbreak ranging from human-to-human transmission to infection monitoring at the community level [91]. In COVID-19 diagnosis, these antibodies’ presence (positive test) or absence (negative test) against SARS-CoV-2 antigens is widely diagnosed using—enzyme-linked immunosorbent assay (ELISA), lateral flow immunoassay (LFIA), and ELISpot.

#### 2.7.1. LFIA

LFIA is also called a lateral flow test (LFT). This is a portable device and considered a POC immunodiagnostic test (Table 1). The test procedure is conducted to reveal the outcome at or near the site of the patient. It works on the principle of rapid immunochromatography, wherein a test strip is conjugated with IgM or IgG or both [92]. A positive test indicates that the person is infected with SARS-CoV-2, and a negative test indicates that he has recovered from COVID-19 [93].By dripping a few drops of whole blood clinical sample (blood + few diluting liquids called buffer), if it contains viral antigens, it will bind against the SARS-CoV-2 antibodies present in the control and assay lines of the test strip. Based on the type of antibodies present in the blood sample of the testing individual 2–3 lines of color band formation is noticed. When compared to the RT-qPCR detection, this test’s diagnostic specificity and robustness were found to be higher [94].

#### 2.7.2. ELISA (Enzyme-Linked Immunosorbent Assay)

Among the various ELISA procedures, indirect ELISA is commonly used test platform, which comprises 96 testable antigen-impregnated microtiter wells (e.g., the spike protein of SARS-CoV-2). These antigens are customized to bind with specific antibodies (IgM or IgG) present in a COVID-19 patient’s serum sample. In the process of detection, a highly specific antigen–antibody reaction complex is formed. Following washing, such a complex combine with the conjugate (antihuman IgG with horseradish peroxidase) and the substrate (3,3′,5,5′tetramethylbenzidine) to give a color change that is readily detected by theELISA’s palate reader. A positive test signal results when the anti-COVID-19 IgG becomessandwiched between the anti-human IgG probe and absorbed antigen. Due to the merit of robust infection stage detection for antigen led to widespread use of the ELISA test to evaluate the infection status in the ongoing COVID-19 pandemic [95].

#### 2.7.3. Enzyme-Linked Immunospot (ELISpot)

It is an antigen-specific T-cell functional test that assess cellular immunity by counting the number of T-cellresponses, which has the ability to produce specific cytokine-interferon *γ* [29,96]. During the process of SARS-CoV-2 infection, T cells are found to play a critical role in cellular immunity by providing long-term protection [97]. During the acute phase of infection, these T cells fight against the virus, among which some of them are transformed into SARS-CoV-2 specific memory cells, which restores the immunological memory needed to prevent future infections. This can be attributed to why people are protected during the second or other times of COVID-19 infection or post-vaccination [98]. This assay consists of microwell plates that are pre-coated with specific antibodies in a dilution culture medium of approximately100 µL per well, after whichthe addition of peptides of viral antigens and peripheral blood mononuclear cells (PBMCs) stimulate the required T-cell-mediated cytokine production. These cytokines accumulate around the T cells and appear as spots that are finally scanned and analyzed through the special immune-spot software [99]. Thus, the ELISpot method has viability to assess the cellular immunity status of an individual during the acute phase of COVID-19 infection.

### 2.8. Neutralization Assay

It is a test for determining the threshold levels or quantitative capabilities of a clinical sample to induce neutralizing antibodies (NABs), which is a measure of humoral response. These NABs confer protective immunity against a disease condition [100]. In addition, the results of this assay are used to correlate clinical sample NABs titers response under a particular medical condition or immunization efficacy study against COVID-19 [101,102]. This assay has also been used to quantify cultured cells capabilities of producing NAB’s titers in 1–2 days or clinical samples (i.e., blood, serum, or plasma) response in hours. In infected cell cultures, NABs are known to directly interfere with viral binding (SARS-CoV-2) to prevent its entry and viral replication [29].NABs generally bind to capsid proteins of the non-enveloped viruses and glycoproteins of the enveloped viruses not only preventthe entry of the virus but also hinder conformational changes. This leads to the formation of pathogen–antibody complexes, which are phagocyted by the macrophages [53]. The latest advancements in this assay have reduced the time of detection from days to hours with the increased feasibility of testing viral disease NABs and vaccine efficacy evaluation [100].

**Figure 6:**
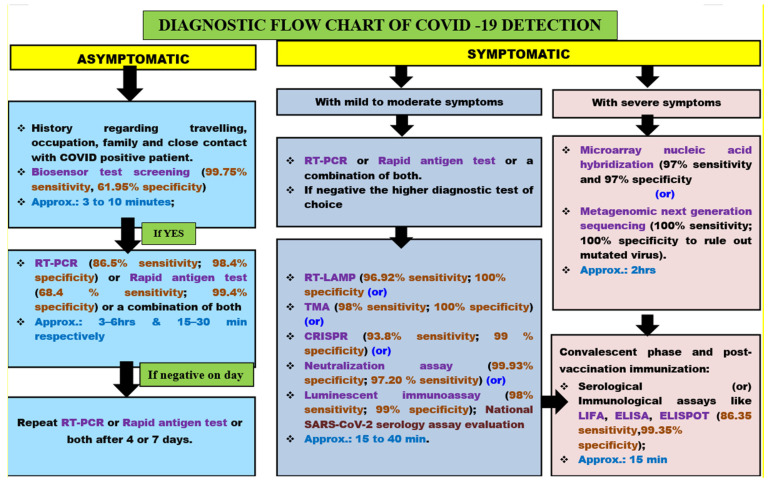
Diagnostic flow chart for COVID-19 disease detection.The probable flow chart used to rule out positive or negative test results forSARS-CoV-2infections in asymptomatic and symptomatic individuals with COVID-19 [103,104,105,106,107,108,109,110,111,112,113].

The probable flow chart of diagnostic tests used to rule out COVID-19 infections in asymptomatic and symptomatic persons has been illustrated in Figure 6.

### 2.9. Rapid Antigen DetectionTest (RADT)

**Figure 7:**
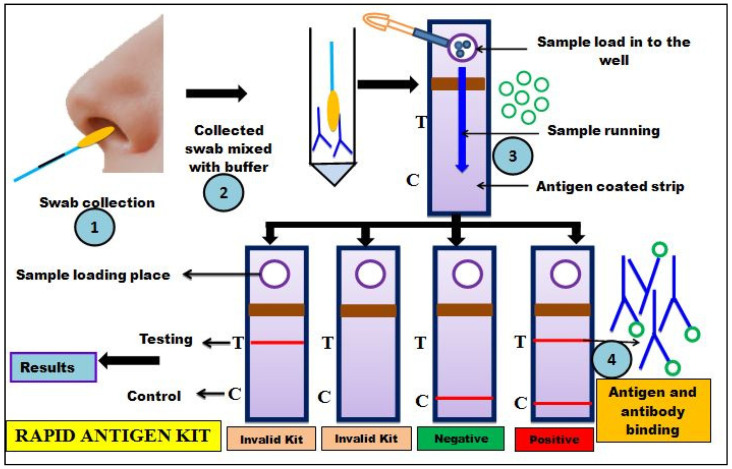
Rapid antigen test (RAT) kit-based detection of SARS-CoV-2 infection: (**1**) test samples are collected in the form of swabs; (**2**) mixing the collected swab samples with buffer; (**3**) loading collected sample into the well of the antigen-coated strip; (**4**) formation of one band representsa negative test, and two bands represent a positive test for COVID-19 infection.

**Figure 8:**
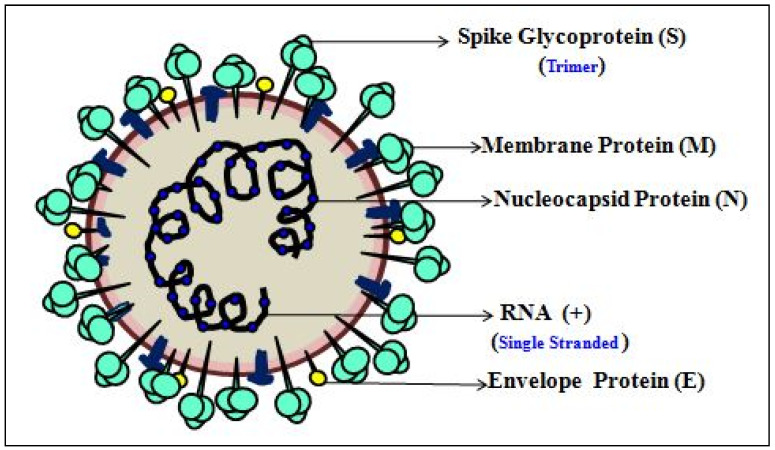
Structure of the SARS-CoV-2 virus. The structure of the COVID-19 virus contains the spike glycoprotein (SP), membrane protein (MP), nucleocapsid protein (NP), an envelope protein (EP), and single-stranded +RNA as the genome.

RADTs are commonly used in situations where molecular detection technologies are unavailable, and they are primarily used for disease testing with symptomatic individuals (Figure 7). These detection methods are simple and portable, which are based on quantitative measurements of antigenic (Ag) surface proteins in terms of either their absence (negative result) or presence (positive result) [54]. Antigen (Ag) is a foreign or pathogenic molecule that readily binds to a B-cell Ag receptor (BCR) or Ag-specific antibody (Ab). RADT works on the principle of identifying the presence of SARS-CoV-2 antigen in clinical samples using specifically designed monoclonal antibodies. The SARS-CoV-2 viral structure (Figure 8) comprises a spike protein (SP), an envelope protein (EP), a major glycoprotein, a membrane protein (MP), and a nucleocapsid protein (NP), whichare the viral antigens supposed to be detected in the clinical sample for COVID-19 detection [54]. The detection of SP in the sample is the most rapidand accurate for detection of COVID-19, as it can be identified from urine or serum samples during the early stages of infection or 10 days post-infection of asymptomatic cases [114].As the NP is larger in size, a sandwich immunoassay is the most commonly used test for its detection, while MP is the most abundant viral protein used for the detection of COVID-19 disease. In addition, EP is the next abundant smallest viral protein that can used to detect COVID-19 disease [93,115]. If the antigen in question is present in enough concentration in the collected sample, they bind to specific antibodies which are encrypted on the test vial. Thus, within 30 min of duration, the viral antigen detection generates a visually detectable signal with a sensitivity of 34 to 80% [54].

### 2.10. Luminescent Immunoassay

This test assay, based on the phenomenon of chemiluminescence (CL), is a chemical reaction in which electromagnetic radiation is generated in the form of light. The chemiluminescence technique is integrated with immunochemical reactions in the chemiluminescence immunoassay (CLIA), wherein chemical probes similar to other labeled immunoassays (such as FIA andELISA) are used to generate detectable light emission [116]. This assay uses synthetic antigens of the coronavirus SP and NP to measure antibody immune responses using a chemiluminescent analyzer [117]. Variations in the titers of SARS-CoV-2 specific antibodies (IgM and/or IgG) in COVID-19 patients represent the phase (i.e., acute or chronic) of COVID-19 infection status [118,119]. Thus, it is a reliable method with high diagnostic sensitivity to know the immunization status and the epidemiological surveillance [120,121]. Moreover, with the development of peptide-based magnetic chemiluminescence enzyme immunoassay, the Diazyme Laboratories of San Diego has advanced the Diazyme DZ-Lite-based SARS-CoV-2 IgM CLIA detection kit and SARS-CoV-2 Ig G CLIA detection kits, which run on the fully automated chemiluminescence analyzer [122,123]. Similarly, a technique with multiplex chemiluminescent immunoassay was found to detect all antibodies like IgG, IgM, and IgA during serological profiling of both COVID-19-positive asymptomatic and symptomatic patients [55].

## 3. Vaccine Platforms

Edward Jenner’s era of vaccination began with the development of the smallpox vaccine in 1976 [124]. Vaccination is an effective, economical, and the most successful health intervention that saves millions of lives every year. Vaccines are biological preparations of antigenic agents that trigger acquired immune responses in order to avert infectious diseases. Vaccine development is a tedious and complex process; it takes time to develop huge amounts of viruses on a wide scale, and it needs level 3 biosafety facilities fortheir production [38]. Vaccine development process often takes 10 to 15years, with a poor success rate [125]. Breakthrough vaccine development platforms are currently being advanced in an unthinkable time frame (12–18 months) with the hope of putting an end to the SARS-CoV-2 pandemic or its future outbreaks/surges [126]. Scientific communities across the world have been consistent in designing different platform-based vaccine candidates to curtail COVID-19disease asearly as possible [39]. Immunization against COVID-19 might be a rightful hope for ending the current epidemic, as vaccine-induced immunity is considerably more likely to confer adaptive immunity against the natural infection process or recurrent SARS-CoV-2 infections [127]. Vaccination hopes to provide long-term immunity against the deadly virus by protecting human beings from becoming ill or averting mortality due to the fact of COVID-19 infection. In all countries, vulnerable populations (elderly) are the highest priority for vaccination. The life of people can return to anormal state with contacts, social events, and traveling only after the performance of vaccination to a population proportion (70%) of herd immunity [43,128]. This may be attributed to development of vaccine-based immunological memory in people’s bodies, which can effectively tackle the present form of the virus and possibly against the mutated virus. If any of these vaccines become less effective against a new form of virus, then one has to change the vaccine’s composition to protect the life of people from new variants of COVID viruses. To eliminate the COVID-19 disease completely from the people, it is necessaryto collect and analyze data continuously on new variants of the COVID-19 virus. Moreover, in future, second, or third generation vaccines having antigenic preparation beyond the SARS-CoV-2 S protein or multiple antigenic targets might be worthwhile in effectively tackling and completely eradicating the SARS-CoV-2 virus from the human population.

**Figure 9:**
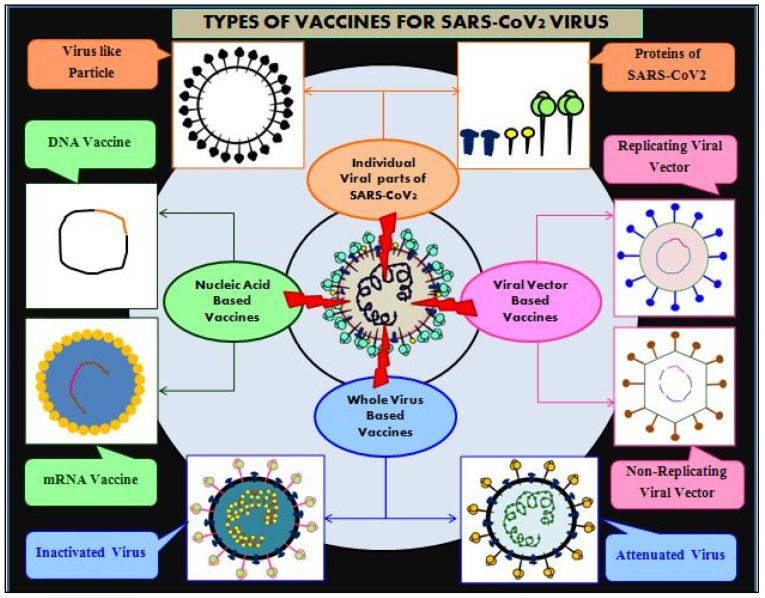
Various types of vaccine platforms used to mitigate COVID-19 pandemic. Major types of vaccine platforms: viral vector vaccines of non-replicating andreplicating types; nucleic acid vaccines based on DNA and mRNA; vaccines based on recombinant proteins/subunit/VLPs virus-like particle;vaccines based on virus-based on inactivated and live attenuated viral components.

As per the currently available data, 14 different vaccines against SARS-CoV-2 have been known to clear phase IIIclinical trials and be approved for worldwide massive and accelerated immunization campaigns to mitigate COVID-19viaEUA [40,129,130]. This is more likely to combat the mounting COVID-19 cases and deaths worldwideurgently. COVID-19 vaccine candidates developed come under different types of vaccine platforms (Figure 9):

1. Viral vector vaccines (non-replicating and replicating);
2. Nucleic acid vaccines (DNA and mRNA);
3. Vaccines based on recombinant proteins (subunit and VLPs virus-like particle);
4. Virus-based (inactivated and live attenuated).

Each type of vaccine candidate has pros and cons in terms of safety, efficacy, and development [35].

### 3.1. Viral Vector Vaccines

**Figure 10:**
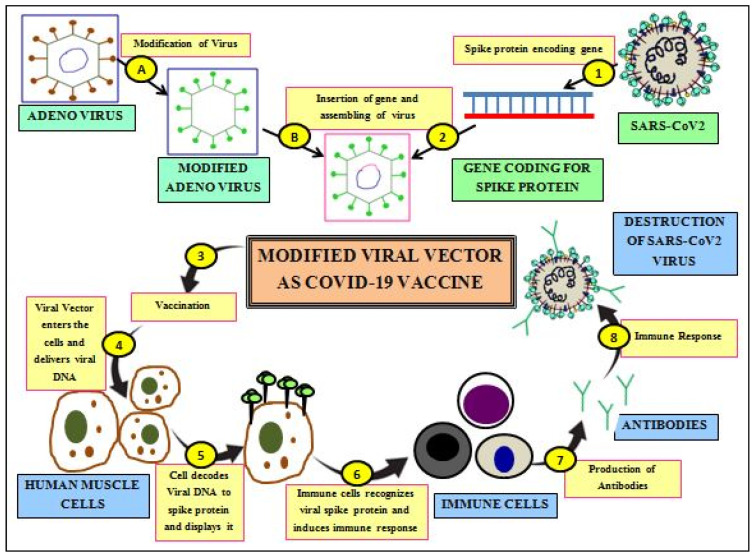
Viral vector-based vaccines for the treatment of COVID-19: (**1**) modification of adenovirus by removing pathogenic genes to make them non-infectious; (**2**) insertion of a gene of interest encoding the SARS-CoV-2 virus’s spike protein into an adenovirus system; (**3**) making a modified adenovirus-based vaccine and their administration into individuals; (**4**) adhesion of a viral vector to human cells and delivery of the antigenic determinant; (**5**) expression of a viral vector antigenic determinant (spike protein) and its display on the surface of human cells; (**6**) recognition of viral spike protein by immune cells; (**7**) immune cells’ production of antibodies (NABs) against viral spike protein; (**8**) immune responses with elicited antibodies neutralize the SARS-CoV-2 virus in vaccinated people.

The viral vector vaccination platform uses non-infectious empty viral particles that self-assemble as infectious virions and having the ability to mimic antigenic (coronavirus) sequences. Due to the lack of a viral genome, these vaccines are empty viral particles of delivery systems containing foreign antigenic proteins (SARS-CoV-2) on their surface. These vaccines use the virus as vectors which are chemically destabilized to make them non-infectious in nature. Due to the natural tendency of host cell infection by viruses, viral vector-based vaccines have the highest ability to carry gene transduction [131]. The majority ofhuman cells are easily infected with adenoviral vectors because they have adenovirus cell surface receptors that aid in adenovirus attachment and entry into the cell. Viral vector vaccines are of either non-replicating (inactivation of viral replicating genes) or replication type. Viral vector vaccines (Figure 10) express the antigenic proteins using the protein machinery of the infected cells, which evokes higher intensities of immune responses of both cellular and humoral types [132]. However, possible reversion to a pathogenic type remains a safety concern [133]. Despite this, viral vector-based vaccines are known to be produced rapidly in bulk quantities and need cold-chain requirements during vaccine storage and transportation [41].

#### 3.1.1. Non-Replicating Viral Vector Vaccines

The majority of the current vaccines developed to mitigate COVID-19 come under this category. This type of vaccine is devoid of the genes necessary for replication; therefore, they cannot produce infectious progeny, posing no risk of vaccination infection [134]. These non-replicating viral vector vaccines are genetically altered adenovirus (Ad) vectors that are impaired to carry replication in humans. It is achieved by disarming viral structural proteins within the vector, thus hindering virion assembly underin vivoconditions. But the assembly of vaccine vector requires the missing structural protein from another helper virus or a transgenic host cell. Commonly used non-replicating types of viral vectors are serotype 26 (Ad26) and serotype 25 (Ad25) of human adenovirus, alphavirus, MVA-modified vaccinia virus Ankara, and adeno-associated virus (AAV) [134]. Other viruses which are used as vectors are the modified vaccinia Ankara (MVA) virus, influenza virus, human parainfluenza virus, and Sendai virus [135,136]. One major drawback associated with these vectors is limited vaccine efficiency due to the pre-existing immunity. However, it is circumvented by using vector types that are either uncommon in human beings [136] or by using viruses that do not induce much immunity or animal-based viruses, such as adeno-associated viruses, are used. Further, pre-existing immunity is circumvented by boosting with one vector or by priming with another vector. Several non-replicating viral-based vaccines against SARS-CoV-2 are in the final stages of clinical tests Due to the replication deficiency, greater doses of these vaccines are required to elicit an immune response. In addition, these vaccines need to be administered in booster doses to confer long-term immunity [40]. Table 2 lists vaccines of this category that were approved for large-scale immunization via EUA, to mitigate the COVID-19 pandemic.

#### 3.1.2. Replicating Viral Vector-Based Vaccines

Unlike non-replicating viral vectors, replicating viral vectors can multiply independently in host cells; hence, a lower dose of this vaccine formulationis adequate to establish protective immunity. These vectors are developed based on attenuated strains of viruses which are modified to express a transgene (e.g., the spike protein of SARS-CoV-2) [51]. Moreover, animal viruses that do not replicate efficiently obviously cause disease manifestation in human beings that are also used to make replicating viral vectors. However, these approach-based vectors undergo multiplication in the vaccinated individuals, which induces a strong immunity in them. There are safety concerns associated with these vaccines due to the pathogenicity of the replicating viral vector vaccines, observed particularly inimmunocompromised individuals. These vectors are often administered intramuscularly or through mucosal routes (oral andintranasal), which may impart immune responses at the specific site of administration. The common replicative types of viral vectors are measles virus (MV), vesicular stomatitis virus (VSV), and adenovirus (Ad-V) [137]. Pertussis, Hepatitis B, and HPV are examples of other vaccines developed comes under this category [138].

### 3.2. Nucleic Acid-Based Vaccines (RNA/DNA)

The nucleic acid (RNA/DNA)-based vaccine represents a novel and quick low-cost-based strategy used to impart protective immunity against SARS-CoV-2 infections. In the history of vaccine development, this is the first time of using this strategy to obtain an approved vaccine (COVID-19 vaccines) in public health programs [139,140]. In this approach, DNA or RNA molecules are engineered to encode antigenic proteins for triggering specific immune responses [141]. Recombinant technology uses nucleic acid molecules (DNA or mRNA encoding disease-specific antigens) in properly stabilized formulations. These nucleic acid molecules (e.g., RNA) are encapsulated to provide durability during storage and transportation. Thus, DNA and mRNA vaccines are driven into the cells using different techniques such as direct injection or encapsulation in nanoparticle form. In the case of DNA vaccines, nucleic acids are circularized forms of plasmids, which alone stable enough to use in formulations. In contrast, an mRNA vaccine needs to be kept intact for their appropriate mode of action. Thus, nucleic acid molecule-based vaccines generally need grouping with appropriate delivery vehicles such as nano or microparticles. DNA or RNA molecules, once they enter the cell, initiate the synthesis of antigens and display it on the cells surface, which after recognition by the immune cells stimulate specific immune response (antibodies) of both humoral and cellular immunity [142]. Intriguingly, nucleic acid-based vaccines can beproduced on a large scale, enablingtheir quick deployment to prevent pandemics. Because of this, several biotech industries such as Pfizer, BiNTech, and Moderna were actively engaged in the nucleic acid-based development of vaccine candidates against COVID-19 [143].

#### 3.2.1. DNA Vaccines

DNA vaccines are produced based on plasmid DNA; it is ahighly attractive vehicle that readily undergoes transcription and translation to produce one or multiple antigens inside cells [144]. An engineered plasmid DNA vector comprises mammalian expression promoters, RNA processing elements, and gene-specific spike proteins (in the case of vaccines against COVID-19) to express the antigenic determinants in the vaccinated individual [145]. Plasmid DNA is highly stable and can be multiplied in larger quantities using host systems (e.g., *Escherichia coli* and *Bacillus subtilis*), which makes this platform attractive for large-scale production. Moreover, as plasmid DNA is very stable at room temperature, itmakesit easy for their long-term storage and transportation conditions. To make DNA vaccines efficient, they are administered via delivery equipment, such as electroporators, and often these vectors know to impart low immunogenicity [146]. However, the predominant challenge is that these vectors induce the risk of mutations and may readily integrate into the genome of the host cell [132,135]. Takis Biotech, LineaRx, Applied DNA Sciences Subsidiary, and Inovio Pharmaceuticals are among the biotech companies that have adopted DNA-based vaccine platforms for the development of vaccines against SARS-CoV-2 [147]. According to a recent study, DNA vaccine candidates werefound to be effective platforms for combating the coronavirus pandemic; however, they may still need to clear regulatory hurdles before theiravailability to the public [148].

#### 3.2.2. mRNA Vaccines

**Figure 11:**
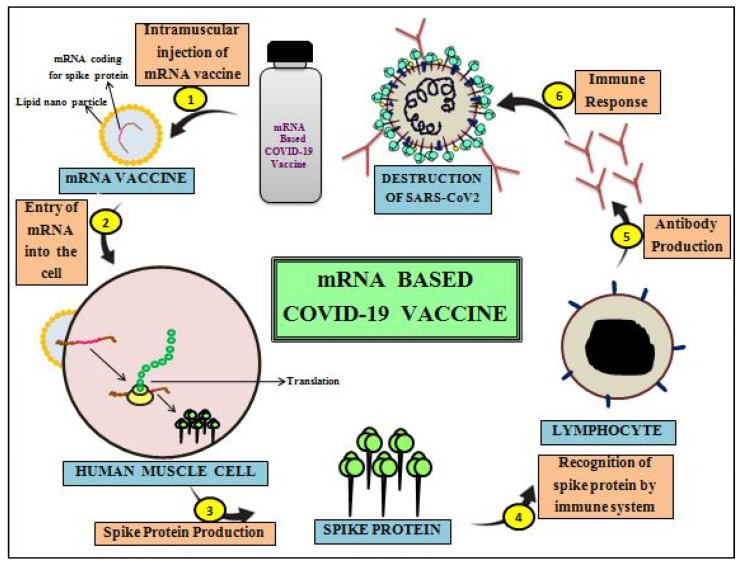
Mechanism of mRNA-based vaccine for developing immunity against COVID-19 infection: (**1**) intramuscular injection of mRNA-based vaccine; (**2**) entry and delivery of spike protein-encoding mRNA into human cells; (**3**) decoding of viral mRNA into spike protein and its display on the surface of human cells; (**4**) recognition of viral spike protein as antigenic determinant by immune cells; (**5**) immune cells’ production of antibodies (NABs) against viral spike proteins; (**6**) immune responses elicit antibodies to neutralize the SARS-CoV-2 virus in vaccinated people.

RNA vectors are relatively recent in terms of development. These vaccines contain mRNA molecules of the single-stranded form, which express genetic information of specific antigen determinant without requiring transcription [149]. When delivered into the cells of the vaccinated individual, the antigenic sequence in mRNA merely needs translation step to make its antigenic determinant (protein). The transcript of mRNA generally encompasses the gene of the interest guarded by 3′and 5′untranslated regions -polyA tail and 7-methylguanosine cap, respectively [150]. The capacity to manufacture vast amounts of mRNA transcripts using conventional methods is merit for the production of mRNA-based vaccines [151]. The objective of particular antigenic expression is served by either mRNA transcripts with modifications (synthetic) encoding the antigenic determinant of interest or self-replicating RNA (Figure 11). The mRNA molecule is naturally unstable due to the fact of its single-stranded (ss) form, and it is readily degraded by the ubiquitous action of ribonucleases. For enhancing the stability of the mRNA vaccine, the mRNA molecules are initially precipitated with 80nmsized liquid nanoparticles (LNPs) andthen packed to injectable forms [141,152]. Phospholipids, PEG, cholesterol, and ionizable cationic lipids make up lipid nanoparticles (LNPs), which assemble to create a stable lipid bilayer that encases the mRNA molecule [153]. The mRNA vaccine candidate is much safer, as it is neither an inactivated virus nor does it possess any protein subunits of the alive SARS-CoV-2 virus.

RNA vaccines, in contrast to traditional vaccines, are a new, potent, and cost-effective platform for against viruses [154]. The mRNA-based vaccine is considered safe, as it cannot integrate into the human/host chromosome [155]. This vaccine platform has not only been rapidly produced, but it has also showed tremendous promise in recent years due to the fact of its nature of simulating a natural kind of infection process through precise antigenic expression in host systems [152,156]. The use of nanotechnology to encapsulate the mRNA with a lipid nanoparticle coating enables its intramuscular delivery much easier [144,151]. Despite this, the RNA vaccine platform is suffering with constraints oflong-term storage and transportation instability as they need stringent cold-chain conditions.

Several RNA vaccines are in development against COVID-19 disease [129]. Among them, a vaccine (mRNA-1273) has been designed based on synthetic viral mRNA, which encodes the entire spike protein of SARS-CoV-2 virus. This is known to have the ability to induce natural infection caused by the naturalSARS-CoV-2 virus. In addition, mRNA vaccines, BNT162b1 and BNT162b2, encode for the entire spike protein and RBD subunit of SARS-CoV-2, respectively. Based on phase III clinical trials data of BNT162b2 after 28 days of its first administration, RNA vaccines showed 95% efficacy against COVID-19. Due to the fact of this, the FDA has granted BioNTech and Pfizer EUA for the BNT162b2 vaccine against COVID-19 [157]. Among the various nucleic acid-based vaccines, Moderna’s mRNA-1273 (SARS-CoV-2 mRNA) vaccine has been successfully tested and utilized for immunization against COVID-19. These features suggest that mRNA-based COVID-19 vaccines are efficient, safe, and good enough in providing immunization in humans. Among the various mRNA-based vaccines, two vaccines of BioNTech/Pfizer and Moderna (mRNA-1273) were provisionally approved for usage in several countries [126]. However, these vaccines are unlikely to induce strong mucosal immunity (oral or nasal) against infectious respiratory pathogens such as SARS-CoV-2, as these vaccines are administered intramuscularly.RNA vaccines were allowedforuse via EUA for large-scale immunization in the process of mitigating the COVID-19 pandemic (Table 2).

### 3.3. Recombinant Viral Protein-Based Vaccines

These are viral protein-based vaccines manufactured by recombinant technologies whichconsist of viral antigenic fragments (immunogens) [158]. They have no genetic materials and are thus comparatively safer compared to whole virus-based vaccines. These are divided into virus-like particle (VLP)-based vaccines, recombinant RBD-based vaccines, and recombinant spike-protein-based vaccines.

**Figure 12:**
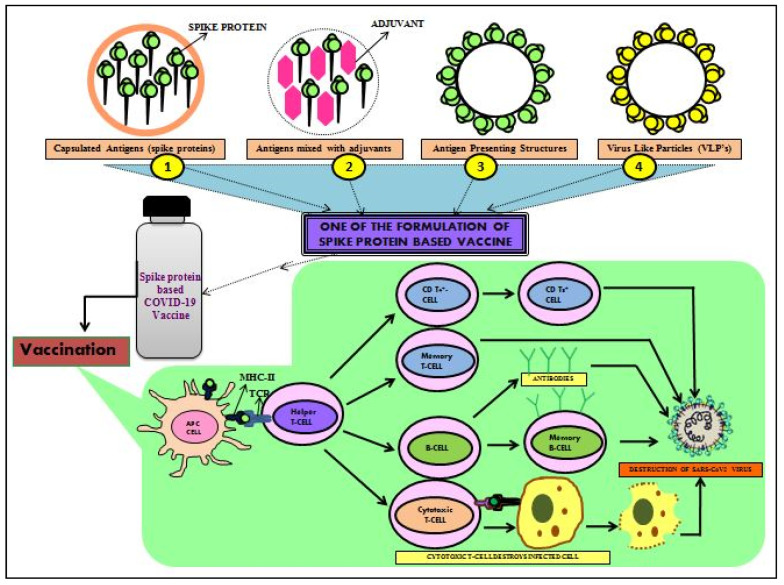
Mechanism of spike protein-based vaccines for developing immunity against COVID-19 infection: (**1**) spike proteins of the SARS-CoV-2 virus is enclosed inside a capsule; (**2**) spike proteins are mixed with adjuvants; (**3**) antigen-presenting structures are made with spike proteins; (**4**) virus-like particles are made with native spike proteins of the SARS-CoV-2 virus. The spike protein-based vaccines arecreated using one of the above four represented protein formulations. Upon vaccination with thespike protein-based vaccine, the antigen-presenting cells recognize the virus’s spike protein and present it to immune cells (i.e., Tcells and Bcells), resulting in both cell and antibody-mediated immunity.

Despite several COVID-19 vaccines having beendeveloped, there is a sufficient global demand for vaccines to control the quick spread of SARS-CoV-2. Some recombinant protein-subunit vaccines against COVID-19 are in the pipeline [87,159]. Protein subunit vaccines are easier to produce, having been made with one or a few harmless proteins or its segments of the pathogen, whose delivery in the host system induces strong host immunity (Figure 12). Insect cells, mammalian cells, yeast, and plants are the model systems used to express recombinant proteins. The extent of given antigenic protein post-translational modifications yields varies depending on the expression system. Most protein subunit-based vaccines require adjuvants to induce enhanced immune responses [142]. Hence, to boost the immune response, this type of vaccine needs adjutants, and also multiple dose administration are mandatory to enhance vaccine efficacy. It is a well-developed platform; existing approved subunit vaccines, viz., HBV and DPT [128]. Most of these vaccines employ either the full length of S protein (vaccines based on recombinant spike protein) or its receptor-binding domain (RBD) as an antigenic determinant (recombinant RBD-based vaccines). The S protein is a surface protein of the SARS-CoV-2 virus that helps in binding the virus to the host cells with the ACE2 receptor for their fusion and entry [10]. Currently, in order to induce enhanced immune responses, different vaccines are produced using the S protein as a vaccine antigenic determinant [160]. Similar to the entire S protein, the RBD fragment induces the neutralizing antibodies (NABs) but lacks other important (neutralizing) epitopes as that of the entire S protein. Thus, vaccines of RBD subunits are not as worthwhile as those of S protein vaccines [38]. At present, different types of recombinant protein-based vaccines are at the stages of preclinical trials. Some RBD-based and spike-protein-based vaccines have entered the clinical trials (Table 2) [129].Clover Biopharmaceuticals Inc., developed a trimerized S protein-based subunit vaccine against COVID-19 [161].

Virus-like particles (VLPs) vaccines are a type of recombinant vaccine made from the antigenic portion of the pathogen, which triggers required specific immune responses. VLPs are generally made with a radius of 20–200 nm, making them ideal for uptake by antigen-presenting cells (APCs) of the host system, thereby eliciting prompt T-cell responses. The interesting feature of these nano-particle-based VLP vaccines is that theyare given as intranasal vaccines spray or inhalers [162]. There are increasing investigations on vaccine development based on nanoparticles [163]. Such vaccines are proposed to have higher specificity, efficiency, and pharmacokinetic properties. But the assembly of the particles is sometimes challenging. Vaccines of this category are under usage against Human papillomavirus and Hepatitis B pathogens [130]. Novavax Inc., developed an S protein-based nanoparticle vaccine (NVX-CoV2373) that was found to be safe and effective incontrolling COVID-19 (Table 2) [164].

### 3.4. Whole Virus Vaccines

**Figure 13:**
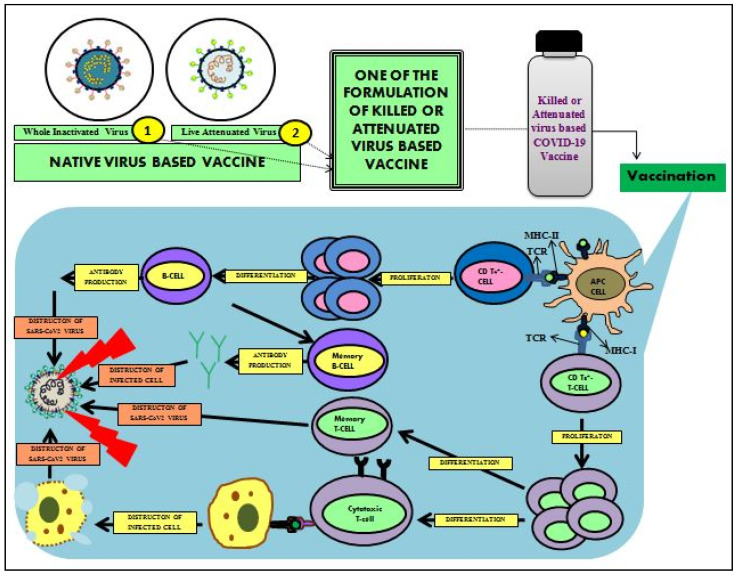
Mechanism of whole virus-based vaccine for inducing immunity against COVID-19: (**1**) whole or native virus-based vaccines are prepared by inactivating the whole virus; (**2**) whole or native virus-based vaccines are also prepared by attenuating the virus. Whole virus-based vaccines aremade with one of the above two represented protein formulations. Upon vaccination of the whole virus-based vaccine, the antigen-presenting cells recognize the inactivated or attenuated SARS-CoV-2 virus and present it to immune cells (i.e., T cells and B cells) to mediate both cell and antibody-mediated immunity.

Live inactivated and attenuated vaccines are the whole virus vaccines with several antigenic components, which induce potentially broad immune responses in the host against the virus. This is the conventional-based vaccine type that forms the basis of many existing vaccines (Figure 13).

#### 3.4.1. Live Attenuated Vaccines

Live attenuated vaccines are developed by using a virus in a weakened form by eitherin vivoorin vitrotechnique or by reverse type of genetic mutagenesis. As a result, the virus replicates to a limited extent, but the virus still has the ability to replicate and mimic immunogenicity similar to natural infection. After their delivery into the host system, these types of vaccines exhibit high immunological efficiency and exert wide cross-protection by inducing humoral, cell-mediated, systemic, and mucosal immunity [165]. The currently available live-attenuated virus-type vaccines include yellow fever vaccine, oral poliovirus vaccine, vaccines against mumps, measles, and rubella [128]. These vaccines need to undergo safety concerns and has tedious process while weakening the viral antigenic components [166]. Currently, a live attenuated type of vaccine for COVID-19 was developed by Codagenix/Serum Institute of India Ltd. (Farmingdale, NY, USA) via codon deoptimization, which is about to roll out for public use.

#### 3.4.2. Inactivated Virus Vaccines

These vaccines consist of live and whole pathogen components in an inactivated or weakened form, which generates an immune response without causing any disease manifestations. These are also called killed vaccines, whose manufacturing iseasy; however, takes a long time for virus culture under biosafety level3 production facilities. These vaccines are actually weakened by subjecting to UV radiation, heat, or chemicals. *β*-Propiolactone is being used as an inactivating agent and additionally need adjutants, such as aluminum hydroxide, for enhanced immunogenicity.This kind of vaccine platform has been widely used over several years as inactivation renders these vaccine formulations safe. Moreover, these vaccines are non-replicating in nature and exhibit no adverse effects even in immune-compromised hosts. The existing inactivated types of vaccines act against seasonal influenza and inactivated polio vaccine, and vaccines against rabies, Japanese encephalitis, and hepatitis-A diseases [128]. When compared to live-attenuated vaccines, these vaccines induce a lower immune response; thus, they need to be given in multiple doses to boost immunity. Despite this, inactivated types of vaccines are highly immunogenic in nature and result in the immune response of the innate type. These vaccines present the whole virus to the host’s immune system, and their immune responses are triggered to matrix, envelope, nucleoprotein, and spike proteins of the SARS-CoV-2 virus.

Some of the important examples of these kind COVID-19 vaccines are Corona Vac from Sino Vac biotech from China (Table 2). Sinopharm of China has produced the BBIBP-CorV vaccine, which has exhibited satisfactory results in clinical tests and proceeded to phase IIItrials in which 79.3% efficacy was shown for against COVID-19. It also has the authorization of conditional marketing (CMA) approval. Another vaccine (BBV152) from Bharath Biotech of India is currently in phase III trials, which has received EUA for itsusage across India and other countries [167].

Table 2 summarizes different COVID-19 vaccine candidates (passed through phase III trials) after EUA, which are widely used for the public in several countries (WHO drafts landscape, 2021).

## 4. The Ideal Next-Generation COVID-19 Vaccines: Mucosal Vaccines and Edible Vaccines

### 4.1. Mucosal Vaccines

Massive efforts have beenmade in thedevelopment of several vaccine candidates as well as their safety testing, immunogenicity levels, host protection levels, and efficacy. Within a few days of a natural SARS-CoV-2 infection or after vaccine administrationprocess in humans, serum neutralizing antibodies are generally produced inpersons [152,168]. Hence, an ideal vaccine forCOVID-19 is anticipated to induce high titers of antibodies that could neutralize the SARS-CoV-2 infection process. These antibodies are known as vaccine-induced neutralizing antibodies (NABs). Further, an ideal vaccine has to reduce non-NABs production by lowering the enhanced respiratory disease (ERD) potential and by minimizing antibody-dependent enhancement (ADE) potential. These potentialities will maintain life-long immunological memory and provide protection against different CoVs [169]. In conjunction, a recent report attributed the correlation of NABsto that of human protection levels from SARS-CoV-2 after COVID-19 infection [170].After natural infection, usually the host’s immune system will induce secretary immunoglobulin A (mucosal antibody of IgA type) and IgG-mediated systemic antibody-mediated immune responses.Secretory IgA protects the upper regions of the respiratory tract, whereas the IgG know to protect the lower respiratory tract [171,172].

Vaccine administration procedures affect a given vaccine candidate’s antigen presentation, expression, immunogenicity, and efficacy. Generally, vaccination administration conductedby parenteral routes includes subcutaneous (SC), intradermal (ID), and intramuscular (IM), whereas mucosal routes include nasal and oral [173]. Since SARS-CoV-2infectshumansviamucosal lines of the respiratory tract [174], vaccine administrationvia the mucosal (oral and/or intranasal) route might be a critical means to prevent disease COVID-19 transmission and prevention [175], because the majority of APCs, especially dendritic cells (DCs), inhabit the mucosal sites and aid in presenting antigen to Tcells, thereby initiatingappropriate immune responses [176]. In addition, IgA immunoglobulin is inhabited at mucosal sites (upper respiratory tract) to prevent pathogenic entry into the body [38]. Most of the current vaccines developed aredelivered intra-muscularly and are known to induce immunity for preventing/attenuating disease and, thus, not staving off viremia or viral shedding from the upper respiratory tract, which perpetuates due to the deprived local IgA-mediated immune responses. Thus, vaccines administered intradermally or intramuscularly induce IgG production without secretory antibodies (IgA). This is not necessarily conferring sterilizing immunity. Sterilizing immunity is a key factor in eliminating viruses and does notcarry any of the viruses. Thus, intranasal vaccine candidates that confer mucosal and/or upper respiratory tract immunity may be rightfully thought to contain the COVID-19 pandemic as the virus neither persists nor allow them to infect othersviaviral shedding. Administration of vaccines through the mucosal/nasal route could effectively prevent infection (via induction of strong mucosal immunity) as the site of infection and mode of transmission of the SARS-CoV-2 type of virus is through the mucosal/nasal site of the respiratory tract [174,177]. Currently, most of the COVID-19 vaccines are administered through parenteral routes; however, a range of mucosal vaccines are in the pipeline to roll out [178,179], which are more likely to evoke a humoral response in oral and nasal mucosa lymphoid tissues and, thus, toughening to prevent upper airway transmission by promoting sterilizing immunity required to combat COVID-19 disease.

### 4.2. Edible Vaccines

Edible vaccines (EVs) are vaccine formulations that humans can eat to protect themselves from viral, parasite, and bacterial infections [180,181]. These vaccines are modified plant fruits or vegetables (edible portions) having specific antigen determinants. EVs are edible parts of modified plants’ fruits or vegetables that are expressed with antigenic determinants (vaccine determinants) at a tissue-specific level, upon their consumption by humans trigger immune responses to protect against a certain illness. The need for such EVs in large scale is increasing due to the mounting number of COVID-19 positive cases across affected countries; hence, there is a pressing need to develop an appropriate vaccine based on edible plant parts [182]. Plant-derived edible vaccines can be a feasible and appealing platform, since they are cost-effective due to the fact of their simplicity of large-scale manufacture and the ability to immunize via the mucosal route. Maintaining the cold chain is a major concern in vaccination technology, as it requires costly and laborious logistics to keep vaccines stable throughout storage and transportation process. EVs may be the best alternative to traditional vaccines because they are easy to use (oral delivery), have no patient hesitancy (as they cover all age groups), and are biofriendly [180,181].

The spike protein of SARS-CoV-2 is the best know antigenic target to be cloned via plant-based vectors and expressed into the plant cells of tomato, spinach, lettuce, and cucumber [182]. Attempts for the production of edible vaccines of COVID-19 were already made in a few plant systems (carrot, tomato, cucumber, and banana) for their large-scale production [181,183]. In addition, various plant-based vaccines have been developed, viz., virus-like particles (VLPs), multi-epitope, and mucosal vaccines [184]. Interestingly, plant-based EVs can be either for nutraceutical purpose or used to curb human diseases [185], deploying CRISPR editing technology [186]. However, several regulatory and technological limitations need to be addressed to make EVs more efficient and applicable. To meet the existing demand for vaccines, especially in the low and middle-income countries (LMICs), these plant-based edible vaccines are viable alternatives and would be the game changer to avert COVID-19pandemics.

## 5. Immunoinformatics in Vaccine Preparation

Immunoinformatics is the science of storing, managing, and analyzing the data related to antigenic variations at the amino acid and genomic levels by comparing the data using computational tools [187]. Numerous immunoinformatics tools, such as AlgPred, VaxiJen server, ToxinPred server, and IEDB immunogenicity, are used to evaluate the allergenicity, antigenicity, toxigenicity, immunogenicity, and interferon-gamma inducing capacity of the viral constructs, respectively [188]. Thus, the information of immunoinformatics is also necessary to predict the exact site of the highest rate of mutagenesis in the spike protein-encoding genes and thereby providing a solution in designing the polyvalent COVID-19 vaccines against the multiple emerging variants of SARS-CoV-19 viruses [189].

## 6. Artificial Intelligence (AI) in the Pandemic Times

The ability of a digital computer to perform tasks commonly associated with intelligence is known as artificial intelligence (AI). AI tools are versatile due to the fact of their application in healthcare management, particularly in COVID-19 pandemic by detecting early COVID-19 diagnosis (with the use of technologies such as machine learning, deep learning, and deep neural network) and also being used for studying Lung Abnormalities to rule out the ARDS from common pneumonia [190]. Currently, AI is used to predict the patient’s need for oxygen therapy andasymptomatic people’s tendency to develop ARDS. This can be ruled out, which is a key clinical symptom representing the severity of COVID-19 infection [191]. Now a day’s deep learning model called COVID-19 detection neural network (CovNet) is used to distinguish between COVID-19 and community-acquired pneumonia. Moreover, AI has implications for COVID-19 coronavirus vaccine redesigning deploying VAXIGN reverse vaccinology and machine language, signifying the versatility of AI to combat COVID-19 [192].

## 7. Conclusions and Perspective

Like other viruses, the quick emergence of different mutating forms of coronavirus is now seen worldwide, which has created havoc in many countries with regard to human health and socioeconomic well-being. An accidental mutation often gives the virus improved transmissibility, and those variants (mutants) become more fit and dominant. The emergence of SARS-CoV-2 variants has caused significant human morbidities and fatalities in the initial days of pandemic due to the lack of our preparation for the rapid spread of the pandemic viruses. This is because that the greatest ability to evade human immunity is due to the decreased neutralizing antibodies against specific variants. These changes in the virus are constantly drifting due to the fact of evolutionary pressures, although more suitable variants will emerge over time. No natural process is ongoing, new varieties may settle and no longer confer the advantage of infectivity and will eventually reach its peak form of transmission. The lessons learned from viral pandemics timelines, which spilled over in humans, showed that all viruses will be stabilized after reaching the most contagious phenotype and will finally become endemic. Albeit, early and precise diagnosis of asymptomatic persons, contact tracing, and timely quarantining of infected persons are the keys to forbiddingfurther transmission of SARS-CoV-2.

To date, there is no definitive clinically approved therapeutics available; hence, the only hope is vaccine intervention to combat outbreak SARS-CoV-2. With the advent of the Edward Jenner vaccinology era, vaccine-induced immunity is only the evidence of strategic protective immunity conferred than that elicited by the natural infection. Thus, to diminish the devastating effects of the SARS-CoV-2 on society, economy, and public health, a safe and effective vaccine is of the highest priority and paramount urgency. The current extraordinary speed with which novel gene-based vaccinations are being developed would quickly put an end to viral replication and spread. SARS-CoV-2 variants are not found to evolve at a level to minimize the acquired immunity conferred by now available COVID-19 vaccines, which roll out via EUA. However, the scientific community should rigorously keep monitoring to promptly diagnose the emergence of “vaccine-piercing”variants and, in that case, rapidly redesign diagnostics and vaccines accordingly using advanced areas of immune-informatics and artificial intelligence.

Additionally, the type (heat-stable vaccines) and route (mucosal-oral andintranasal) of vaccine administration would be viable options to avert the COVID-19 pandemic. Hence, variant-specific updating of diagnostics and vaccines should go hand in hand to come out of this public health emergency. Thus, an optimized COVID-19 immunization provides an expectation for an end to this pandemic disease, with equal access and optimal shots for people of all ages, especially in the world’s most densely populated nations (LMICs). Regardless of political ideologies and socio-sanitary settings, integrative perspectives may not be overlooked. Furthermore, to defeat the invisible enemy, SARS-CoV-2, optimizing the human lifestyle with a balanced diet, adequate sleep cycle, and physical activity are critical. As timing going, more reliable clinic treatments will also be developed for treated patients with COVID infection, which include but are not limited to new pharmaceutical drugs and CRISPR-based diagnosis and treatments using various CRISPR/Cas tools [193]. We believe that humans will eventually win this battle and that all the current chaos will be brought under control.

## Figures and Tables

**Table 1:** Characteristic Features of Various Diagnostic Tests used for Detection of SARS-CoV-2-Based COVID-19.

Number	Type of Diagnosis	Advantages	Disadvantages	Sample Used	Type of Test	FDA Approved Kits	References
1	RT-PCR-based assay	Rapid, cost-effective, more sensitivity, qualitative and quantitative analysis possible.	Technique sensitive, needs enormous expertise, even a small amount of contamination leads to falseresults, needs equipment such as athermocycler.	Nasopharyngeal swab, sputum, bronchoalveolar lavage fluid, stool	Laboratory based	Gravity Diagnostics SARS-CoV-2 RT-PCR Assay, ARS-CoV-2 assay Xpert Xpress SARS-CoV-2 test, COVID-19 RT-PCR test, TaqPath COVID-19 Combo kit, STANDARD M n CoV RT detection kit etc.	[30]
2	Isothermal nucleic acid amplification-based assay (RT-LAMP)	Quick, non-expensive as no thermocycler needed, cost-effective, streamlined method, more sensitivity and specificity.	Difficulty in designing the primers.	Nasopharyngeal swab, sputum, stool	Laboratory based/point of care	iAMP COVID-19 detection kit, ID NOW COVID-19.	[45]
3	Transcription mediated amplification (TMA)-based assay	Quick as it involves rapid kinetics resulting in the production of billion-folds of amplicons within 15–60 min, multiple targets can be amplified simultaneously, no expensive thermocycler is needed.	The in vitro transcribed RNA is susceptible to both enzymatic and chemical degradation.	Nasopharyngeal swab, sputum, stool	Laboratory based/point of care	Cue COVID-19 test	[43,44]
4	CRISPR-based assay	Simplicity and efficiency compared to the other gene targeting technologies.	Genetic instability and diversity.	Nasopharyngeal swab, bronchoalveolar lavage fluid	Laboratory based	CRISPR-based LAMP with lateral flow assay, SARS-CoV-2 DETECTR	[46,47]
5	Genomics sequencing	Diversified microbial organisms can be identified especially in outbreak tracking and infectious diseases including their surveillance and mutations.	Expensive and difficult to analyze the data.	Nasopharyngeal swab	Laboratory based	Illumina COVIDSeq Test	[48]
6	Microarray-based assay	Expression levels of a large number of genes can be measured simultaneously, multiple regions of a genome can be genotyped.	Time-consuming, expensive, technique sensitive, difficult to interpret the data, results are not always reproducible.	Nasopharyngeal swab, bronchoalveolar lavage fluid	Laboratory based	CovidArray	[49,50]
7	Biosensors	Quick, cost-efficient, and sturdy diagnosis.	Improper handling by the non-trained clinical staff gives false results	Nasopharyngeal swab, sputum, stool	Point of care	CANARY biosensor	[31]
8	Serological and immunological-based assays	Rapid, easyto use, post-vaccination immunity monitoring.	Uncertainty in test accuracy.	Serum, plasma, or blood	Laboratory based/point of care	BioCheck SARS-CoV-2 IgG AntibodyTest Kit, EllumeCOVID-19Home Test, OmniPATHTM COVID-19 Total Antibody ELISA Test, Anti-SARS-CoV-2 Rapid Test, DPP COVID-19 IgM/IgG System	[29]
9	Neutralization assay	High sensitivity and specificity.	It does not quantitatively measure the minor antigen and antibody relationship between the strains.	Serum, plasma, or blood	Laboratory based	Svnt KIT, cPassTM kit	[51,52,53]
10	Rapid antigen-based assay	Low cost, easy, rapid, contact tracing, and screening of the huge population possible.	Renders falser positive and false-negative results, which is to be correlated with could symptoms.	Nasopharyngeal swab, sputum, stool	Point of care	STANDARD Q COVID-19 Ag, BIOCARD Pro COVID-19 Rapid Ag test kit, VSTRIP COVID-19 Antigen Rapid Test, CIP test COVID-19 Antigen Card Test, Sofia 2 SARS Ag FIA technology, etc.	[54]
11	Luminescent immunoassay	Good sensitivity, broad dynamic range, and applicable over a reasonably broad spectral range.	Technique sensitive, as it lacks sufficient selectivity and sensitivity to various physicochemical factors.		Serum, plasma, or blood and nasopharyngeal swab	m2000 SARS-CoV-2 assay, AGLUMI IgG/IgM de 2019-nCoV (CLIA)	[55]

**Table 2:** COVID-19 Vaccines Details Enlisted with Developers/Manufacturers, Number of Doses, Efficacy, Storage, Conditional Approval, and Current Stage of Clinical Trials [39,126,127].

Sl.Number	Vaccine Brand	Type	Developers/ Manufactures and Authorization Date for EUA	Number of Doses	Gap Between the Doses	Efficacy andApproval for Age Group	Country	Storage at and Number of Doses per Vial	Storage for	Status
Viral Vector Vaccines (Replicating and Non-Replicating Virus Based Vaccines										
1	Johnson & Johnson (Janssen) (JNJ- 78436735)	Viral Vector (Non replicating Human Adenovirus vector)	Johnson & Johnson 5 March 2021	1	Not Applicable	70–85% Approved for ages 18 and above	Multinational (Netherlands, US, Belgium)	2 to 8 °C 5 Doses per Vial	3 Months	In phaseIII trials
2	Oxford-Astrazeneca (AZD-1222; Covishield)	Viral Vector (Non-Replicating Viral Vector ChAdox1-S)	Oxford University-Astrazeneca 26 February 2021	2	84 Days	70–90% Approved for ages 18 and above	Multinational (UK, Sweden, India)	2 to 8 °C	6 Months	Approved for restricted emergency use in India and UK
3	Covishield	Viral Vector (ChAdox1_nCoV-19)	Serum Institute of India Pte. Ltd. 15 February 2021	2	84 Days	70–90% Approved for ages 18 and above	India	2 to 8 °C	6 Months	Approved for restricted emergency use in India
4	Gamaleya-Sputnik V (rAd26, rAd5)	Viral Vector (Recombinant adenovirus vaccine-rAd26, rAd5)	Gamaleya Research Institute 19 March2021	2	28 Days	85–90% Approved for ages 18 and above	Russia	−18.5 °C (Liquid form) 2 to 8 °C (Dry form)	3 Months	Early use in Russia, emergency use in Belarus and Argentina
5	Ad26.COV2.S	Viral Vector (Recombinant Replication incompetent adenovirus type 26 (Ad26) vectored vaccine encoding the SARS-CoV-2 Spike (S) Protein)	Janssen-Cilag International NV 12 March 2021	2	28 Days	85–90% Approved for ages 18 and above	Belgium	2 to 8 °C	3 Months	Emergency use in Belgium
6	Convidecia	Non-Replicating Viral vector	Cansino Biologicals	1	-	65.3% Approved for ages 18 and above	China	2 to 8 °C	3 Months	Limited use in China
Nucleic Acid Based Vaccines (RNA/DNA)										
7	Pfizer-BioNtech (Comirnaty BNT162b1 & BNT162b2)	mRNA in Lipid nanoparticle	Pfizer BioNtech, Fosum Pharma 31 December2020—WHO Approved	2	21 Days	90–94% Approved for ages 12 and above	Multinational (US, Canada, Mexico & Germany)	−80 to −60 °C 6 Doses per Vial	6 Months	Approved for full use/emergency use in several countries
8	Moderna (mRNA-1273)	mRNA in Lipid Nanoparticle	Moderna, BARDA, NIAID 23 December 2020	2	28 Days	90–94% Approved for ages 18 and above	US	−25 to −15 °C 10 Doses per Vial	7 Months	Approved in Canada and Emergency use in US
9	Cure Vac (CVnCoV)	mRNA	Curevac	2	28Days	90% Under review by EMA	Germany	2 to 5 °C	3 Months	Limited use in Germany
10	INO-4800	DNA Vaccine	Inovio Pharmaceuticals + International Vaccine Institute + Advaccine (Suzhou) Biopharmaceutical Co., Ltd.	2	28 Days	Advanced stage of Development	China	Room Temperature	More than One year	In phaseIII trials
Protein Based Vaccines										
11	Novavax (NVX-CoV2373)	Recombinant Nanoparticle (Spike glycoprotein+ Matrix-M Adjuvanted)	Takeda Pharmaceutical Company, Japan Serum Institute of India	2	28 Days	85–90% Approved for ages 18 and above	Multinational (US, India)	2 to 8 °C	3 Months	In phaseIII trials
Whole Virus Vaccines (Live Attenuated and Inactivated Virus Vaccines)										
12	Sinovac (CoronaVac)	Whole-Inactivated Virus (Formalin Inactivated + Alum adjuvanted)	Sinovac Biotech-China 22 February 2021	2	3–4 Weeks	60–75% Approved for ages 18 and above	China	2 to 8 °C	12 Months	Limited use in China
13	Bharath Biotech Covaxin (BBV152)	Whole-Inactivated Virus	Bharat Biotech—ICMR 19 April 2021	2	28 Days	70–80% Approved for ages 18 and above	India	2 to 8 °C	3 Months	Approved for restricted use in emergency situation in India
14	Vaccine- Covilo/BBiBP-CorV/BiBP	Inactivated Virus	BBIBP by Sinopharm- Beijing Institute of Biological Products & BIBP by Sinopharm- Wuhan Institute of Biological Products 7 May2021	2	28 Days	79.3% Approved for ages 18 and above	China	2 to 8 °C	3 Months	Approved in UAE, China, and Bahrain

## Data Availability

Not applicable.
